# Citation Patterns of a Controversial and High-Impact Paper: Worm et al. (2006) “Impacts of Biodiversity Loss on Ocean Ecosystem Services”

**DOI:** 10.1371/journal.pone.0056723

**Published:** 2013-02-20

**Authors:** Trevor A. Branch

**Affiliations:** School of Aquatic and Fishery Sciences, University of Washington, Seattle, Washington, United States of America; University of Kent, United Kingdom

## Abstract

Citation patterns were examined for Worm et al. 2006 (*Science* 314∶787–790), a high-impact paper that focused on relationships between marine biodiversity and ecosystem services. This paper sparked much controversy through its projection, highlighted in the press release, that all marine fisheries would be collapsed by 2048. Analysis of 664 citing papers revealed that only a small percentage (11%) referred to the 2048 projection, while 39% referred to fisheries collapse in general, and 40% to biodiversity and ecosystem services. The 2048 projection was mentioned more often in papers published soon after the original paper, in low-impact journals, and in journals outside of fields that would be expected to focus on biodiversity. Citing papers also mentioned the 2048 projection more often if they had few authors (28% of single-author papers vs. 2% of papers with 10 or more authors). These factors suggest that the more knowledgeable the authors of citing papers were about the controversy over the 2048 projection, the less likely they were to refer to it. A noteworthy finding was that if the original authors were also involved in the citing papers, they rarely (1 of 55 papers, 2%) mentioned the 2048 projection. Thus the original authors have emphasized the broader concerns about biodiversity loss, rather than the 2048 projection, as the key result of their study.

## Introduction

In a single month, November 2006, two papers appeared that created a controversy over the influence of the journals *Science* and *Nature* on fisheries research. The first of these two papers, “Faith-based fisheries” by Ray Hilborn [1] in *Fisheries*, argued that a long string of papers published in *Science* and *Nature* (namely [2]–[6]) had been selected for publication not because of their scientific merit but because of their publicity value. Hilborn [1] argued that it was only because these papers focused on the decline and collapse of fisheries that they appeared in *Science* and *Nature*, and that the system of peer review had failed to detect substantial flaws in each of these papers. The second paper appeared in *Science* in the same month, “Impacts of biodiversity loss on ecosystem services” by Boris Worm and others [7]. Their paper demonstrated through a large body of evidence that biodiversity loss greatly reduces the ecosystem services that we obtain from the oceans, and also contained an analysis projecting “the global collapse of all taxa currently fished by the mid–21st century (based on the extrapolation of regression in Fig. 3A to 100% in the year 2048)”. This projection of global seafood collapse by 2048 was highlighted in their associated press release [8]. The press release resulted in prominent coverage of the projection in major news outlets, and provoked a reaction from some fisheries researchers [8] and 10 rebuttals [9]–[18], including three rebuttals by Ray Hilborn [10]–[12].

At this point a remarkable turn of events occurred, as told best by Stokstad [19]. The chief protagonists Boris Worm and Ray Hilborn met for a National Public Radio interview and decided that a public controversy served the interests of neither the scientific community nor the public. They invited about 20 prominent scientists and dozens of graduate students to join in a collaborative project facilitated by the National Center for Ecological Synthesis and Analysis (NCEAS), with the aim of compiling new datasets to reach a consensus view of the state of the world’s fisheries. The resulting analysis showed that although 63% of assessed fisheries are below the biomass that would produce maximum sustainable yield, harvest rates are now at or below sustainable levels in 7 of 10 well-studied ecosystems [20]. These lower harvest rates should promote rebuilding to biomass to levels that would support maximum sustainable yield.

Following some passage of time since the original controversy over the 2048 projection in Worm et al. [7], it is worth taking a retrospective look at this period in fisheries science through the lens of scientific impact, as revealed by six years of citations. Despite the controversy, or perhaps because of it, Worm et al. [7] is a highly cited paper (799 citations at 20 January 2013 in the Web of Science), and was among the top 10 most cited fisheries papers in 2012 [21]. Past citation analysis shows that the rebuttals to the 2048 projection in Worm et al. [7] had virtually no effect on citation rates or the impressions that people gained about this paper [22]. This paper expands on that citation analysis, to address three main questions:

1. Did the framing of the press release result in a greater frequency of citations mentioning the 2048 projection?
2. Do citations of Worm et al. [7] mostly concern biodiversity and ecosystem services, fisheries collapse, or the 2048 projection?
3. Do the original coauthors of Worm et al. [7] believe the 2048 projection to be valid, despite the rebuttals and controversy?

## Materials and Methods

A list of 664 papers citing Worm et al. [7] was obtained from the ISI Web of Science on 29 July 2012, and an electronic file of each was obtained over a period of months. Most electronic files were obtained through the University of Washington’s online library (N = 622) or had previously been obtained (N = 26). The remaining papers (N = 16) were requested from the corresponding authors, or obtained through interlibrary loans. For each citing paper, all citations of Worm et al. were found, the text surrounding each citation was extracted, and the location of the citation within the text was noted (i.e., first sentence, first paragraph, last paragraph, abstract, introduction, methods, results, discussion, conclusions).

### Word Frequencies

To assess whether the framing of the press release influenced subsequent citations, three word clouds were created using the online software package Wordle (www.wordle.net): from the text in Worm et al. [7] itself; the text in the accompanying press release; and the text surrounding all citations of Worm et al. [7]. To create each word cloud, all of the text was copied into a single document, and then author names, journal names, and institutions were deleted, as were references to figures, tables, and websites. Acronyms were spelled out except for LMEs (large marine ecosystems) and MPAs (marine protected areas). Phrases citing references other than Worm et al. [7] were excluded, and in a few cases phrases from previous sentences were inserted to replace words such as “they” that referred back in the text. Singular forms of common words were replaced with plurals (e.g., fisheries, collapses, ecosystems, declines), and words beginning with upper case letters were replaced with lower case letters. In addition to creating word clouds, word frequencies were calculated for the most commonly used non-trivial words, in addition to the less commonly used words “collapses”, “2048”, and “2050” (to track the frequency of citations referring to the 2048 projection).

### Assessing how Citing Papers Referred to Worm et al

The extracted words surrounding citations of Worm et al. [7] were assessed to determine whether the citing paper referred to the relation between biodiversity and ecosystem services (including the relation between biodiversity and either productivity or resilience); to fisheries collapse (citing text had to specifically mention fisheries); or to the 2048 projection (i.e., mentioning 2048, 2050, or mid-century plus fisheries collapse). It was possible for a given citation to be classified in zero, one, two, or all three of these categories. Note that all citations mentioning the 2048 projection were also classified as concerning fisheries collapse.

### Characteristics of Citing Papers Referring to Different Aspects of Worm et al

To assess whether the original coauthors of Worm et al. [7] cite the 2048 projection, each citing paper was classified as either sharing or not sharing an original coauthor. For each citing paper the following additional characteristics were also gathered: gender, institution and country of the first author; journal name; journal impact factor (not available for 13 papers); general field of the journal; year of publication; total number of authors; and number of times that Worm et al. [7] was cited within the paper.

Of these attributes, gender required some sleuthing. Where gender was not obvious it was inferred from the website Baby Name Guesser (http://www.gpeters.com/names/baby-names.php) which returns the ratio of male to female babies born with a particular name. Where the citing reference only listed the initials of the first author, a search was made for the full name by looking for other papers by the same author and examining the author’s web page. Using these methods, gender was obtained for all but two citing papers.

These attributes of the citing papers were then used to explain whether the citing papers referred to biodiversity and ecosystem services, fisheries collapse, and/or the 2048 projection. Chi-square contingency tables were used to determine whether citation frequencies within each category (e.g., gender) differed from frequencies expected by random chance. P-values were computed using a Monte Carlo simulation test with 100,000 random samples, as implemented by *chisq.test* in R [23].

## Results

### Word Frequencies

**Figure 1:**
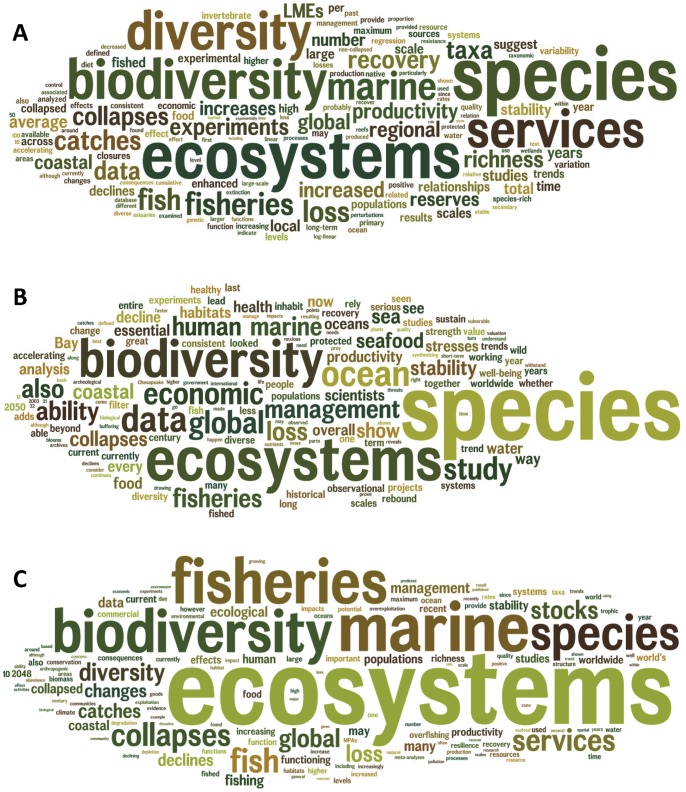
Frequency of words in the original paper, press release, and subsequent citations. Word clouds showing the relative frequency of words (A) in Worm et al. [7], (B) in the press release associated with Worm et al., and (C) in the text surrounding all citations of Worm et al. (bottom picture). Created using www.wordle.net.

Commonly used words in Worm et al. [7], their press release, and in subsequent citation texts are “species”, “ecosystems”, “biodiversity”, “marine”, “ocean”, “services”, “fish”, and “fisheries”; while “2048” (and its variants “2050” and “mid-century”) were less commonly mentioned (Fig. 1, Table 1). Compared with the original paper, the press release referred much less to “services”, “fish”, and “collapses”, but referred more to “ocean” and “2048”. The 2048 projection is further emphasized in the press release by the main heading “Accelerating loss of ocean species threatens human well-being”, subheading “Current trend projects collapse of all currently fished seafoods before 2050”, and an illustrative figure devoted to the 2048 projection. Likely in consequence, the citing papers include mentions of “2048” and its variants four times more often than the text of the original article. The citing papers also refer more to “fisheries” and “ecosystems”, and refer less to “species” and “services” (Table 1). Thus, there is some evidence that increasing the prominence of the 2048 projection in the press release resulted in citing papers referring to this projection more often than expected.

**Table 1:** Frequency of common words (number per 1000 words) in Worm et al. [7], in the press release for Worm et al., and in sentences citing Worm et al. in other papers.

Words	A. Worm et al. (2006)	B. Press release	C. Citation text	Ratio B/A	Ratio C/A
Species	18.40	18.73	10.59	1.02	0.58
Ecosystems	13.90	12.49	20.43	0.90	1.47
Biodiversity	11.45	11.45	11.75	1.00	1.03
Marine	8.18	5.20	12.96	0.64	1.58
Ocean	3.27	11.45	3.78	**3.50**	1.16
Services	11.04	1.04	6.06	**0.09**	0.55
Fish	6.54	2.08	6.93	**0.32**	1.06
Fisheries	6.54	5.20	12.33	0.80	1.89
Collapses+collapsed	9.40	4.16	9.18	0.44	0.98
2048+2050+ mid-century	0.82	2.08	3.24	**2.55**	**3.96**
Total word count	2446	961	24082		

The ratios of word frequencies in the latter two sources, compared to Worm et al., are also given; boldface text indicates where these ratios are less than 0.5 or more than 2.0.

### Assessing how Citing Papers Referred to Worm et al

Papers citing Worm et al. [7] were published in a wide range of journals (N = 242, most frequently in *PLoS ONE* and *Marine Policy*, each accounting for 5% of all citations), spanning many fields. First authors were often male (71%) and resided in 42 countries, most frequently the USA (37%) and Canada (13%). Within the citing papers, Worm et al. was cited most often in the introduction (62%) and discussion (44%), a surprising number of citations occurring in the first paragraph (46%), first sentence (20%), or last paragraph (7%). Citations were seldom situated in the abstract (1%), methods (3%) or results (<1%).

Relatively few citing papers mentioned the 2048 projection (N = 73, 11%) compared with those that mentioned fisheries collapses (N = 262, 39%), or the relationship between biodiversity and stability (N = 267, 40%); and 28% did not refer to any of these three topics, instead mentioning other topics in the original paper such as marine protected areas, biodiversity in general, and ecosystem degradation.

### Characteristics of Citing Papers Referring to Different Aspects of Worm et al

**Figure 2:**
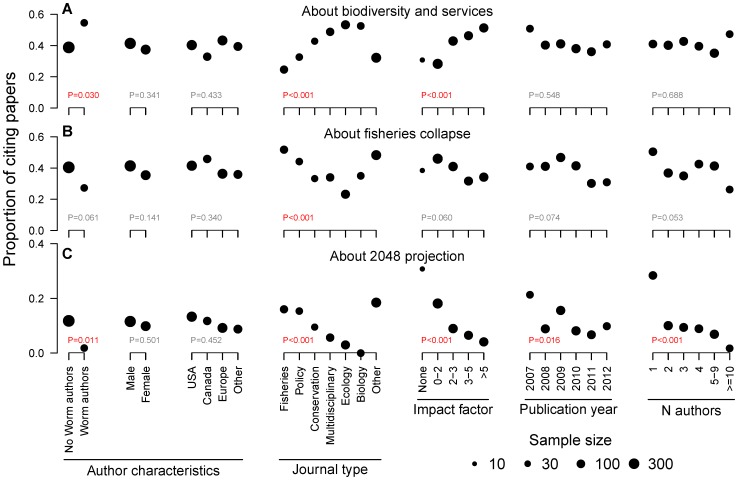
Citation patterns of papers citing Worm et al. [**7**] **.** The proportion of papers citing Worm et al. [7] that referred to (A) the relation between biodiversity and stability or ecosystem services, (B) fisheries collapse, or (C) the projection that all fisheries would be collapsed by 2048. Circle sizes are proportional to the number of articles. The P-values from a chi-square contingency test are shown in the lower left portion of each panel. Author gender and author country represent first authors.

There were some clear patterns in which types of citing papers mentioned the 2048 projection (Fig. 2C). The original coauthors of Worm et al. almost never mentioned the 2048 projection (1 of 55 papers, 2%), whereas citing papers containing no coauthors of Worm et al. did so more frequently (72 of 609 papers, or 12%). The probability of observing such a great difference or greater by chance is P = 0.011. The only case where a coauthor of Worm et al. referred to the 2048 projection was in a paper with Ben Halpern as second of four authors [24], but in nine other papers with Ben Halpern as a coauthor, the 2048 projection was not mentioned. The type of journal influenced how Worm et al. was cited. The 2048 projection was referred to most often by journals in the “other” category, followed by fisheries and policy journals, and was referred to only infrequently by ecology and biology journals. Two other strong patterns emerged: the lower the impact factor of the journal, the more frequently the 2048 projection was cited; and the fewer authors there were on a paper, the more frequently the 2048 projection was cited. This latter pattern is particularly strong: 27 of 95 (28%) single-authored papers mentioned the 2048 projection, compared with 1 of 57 (2%) papers with 10 or more coauthors. Citing papers published soon after Worm et al. also tended to refer to the 2048 projection more often, whereas gender and country of the first author had no statistically significant impact.

Citing papers referring to fisheries collapses did not have many clear explanatory factors (Fig. 2B); no statistical significance (at the 5% level) was associated with whether the authors were shared with Worm et al. [7], author gender or country, journal impact factor, or publication year. However, journal type did play a role: fisheries, policy, and other journals mentioned fisheries collapse more often than ecology journals; and the fewer the number of authors, the more often fisheries collapse was mentioned.

Citing papers referred to the relation between biodiversity and ecosystem services in the opposite manner to those noted for the 2048 projection or fisheries collapse. Biodiversity and ecosystem services were referred to more often by ecology and biology journals and by high-impact journals (Fig. 2A). In addition, coauthors of Worm et al. [7] more often mentioned biodiversity and ecosystem services (30 of 55 papers, 55%) than citing papers lacking Worm et al. coauthors (237 of 609 papers, 39%).

## Discussion

Worm et al. [7] has become a must-read classic paper for marine researchers, as well as a case study in translating science for the media [25]. The press release headlined the most newsworthy part of the paper: the projection that all currently fished seafood would be collapsed by 2048 if current trends continue, resulting in citing papers mentioning this projection four times more often that would be predicted from word frequencies in the original paper. However, only 11% of all citations referred directly to the 2048 projection, compared with 39% mentioning fisheries collapse in general, and 40% commenting on the relation between biodiversity and ecosystem services. Thus, although the press release may have increased citation patterns somewhat, many more citing papers still referred to the main point of the paper about biodiversity and ecosystem services.

The key characteristic of papers that referred to the 2048 projection was related to the familiarity of authors with the controversy over this projection. At the one extreme, coauthors of the original paper, who would have been acutely aware of the controversy, only once mentioned the 2048 projection in their own papers out of 55 papers in which they cited Worm et al. Similarly, papers with many authors were also very unlikely to refer to the 2048 projection (1 of 57 papers with 10 or more authors), perhaps because this increased the probability that one or more of the authors would be aware of the controversy. Additionally, papers published in journals with high impact factors, and presumably reaching a larger and more general audience, were less likely to refer to the 2048 projection–this was mentioned in only 4% of papers with an impact factor greater than 5.0, but 18% of papers with an impact factor less than 2.0. One possibility is that low-impact journals were trying to court controversy to increase their impact factors. Finally, although it would be expected that most citations of Worm et al. [7] would be in journals covering biology, conservation, policy, fisheries, ecology, or multidisciplinary areas, a surprising number of citing papers (211, 32% of all papers) belonged in other categories. These “other category” papers often referred to the 2048 projection (18%), particular when published in journals covering fields that would be relatively unfamiliar with the fisheries literature; for example, journals in the fields of business, religion, epidemiology, nutrition, socialism, genomics, ethics, and technology.

Similar characteristics explained patterns in which citing papers referred to biodiversity and ecosystem services, but here the patterns were reversed in a striking mirror-image pattern in Fig. 2. This can be explained by the divergent interests of authors and editors in different disciplines together with the tendency of most papers to focus on a single topic when citing Worm et al. Coauthors of Worm et al., papers in high impact journals, and papers in ecology and biology journals were significantly more likely to mention biodiversity impacts on ecosystem services, whereas gender, country, year and number of authors were not significant factors in explaining these patterns.

### Conclusions

These results suggest that press coverage does influence how papers are cited, but despite this influence, only 11% of citing papers referred to the 2048 projection. Papers that mentioned the 2048 projection had characteristics that suggest unfamiliarity with the controversy surrounding this projection, namely papers with few authors, published in journals with low impact factors, in fields far removed from ecology and fisheries, and sharing no coauthors with the Worm et al. paper. For such papers, it is easy to see how the authors, editors, and reviewers could be unaware of the controversy over the 2048 projection. Most interestingly, the original coauthors of Worm et al. only once out of 55 occasions mentioned the 2048 projection in subsequent citations of their own paper.

## Supporting Information

Table S1 **Complete list of papers examined, citation texts referring to Worm et al.** [**24**] **, and coded variables used in the analysis.**
(XLSX)
